# Alterations of gut microbes and their correlation with clinical features in middle and end-stages chronic kidney disease

**DOI:** 10.3389/fcimb.2023.1105366

**Published:** 2023-03-24

**Authors:** Hao Chen, Jingyan Wang, Qin Ouyang, Xinyue Peng, Zheng Yu, Jianwen Wang, Jing Huang

**Affiliations:** ^1^ Department of Parasitology, School of Basic Medical Science, Central South University, Changsha, China; ^2^ Department of Microbiology, School of Basic Medical Science, Central South University, Changsha, China; ^3^ Department of Nephrology, The Third Xiangya Hospital, Central South University, Changsha, China

**Keywords:** chronic kidney disease, end-stage renal disease, gut microbe, community composition, clinical indicators

## Abstract

Gut microecosystem has been shown to play an important role in human health. In recent years, the concept of the gut-kidney axis has been proposed to explain the potential association between gut microbiota and chronic kidney disease (CKD). Here, a cohort of fecal samples collected from patients with CKD (n = 13) were involved. The composition of gut microbial communities and clinical features in CKD and end-stage renal disease (ESRD) were characterized. Our study focused on the changes in gut microbiome and the correlation with clinical features in patients with CKD and ESRD by analyzing high-throughput sequencing results of collected feces. We elucidated the alterations of gut microbiota in CKD patients at different stages of disease and initially identified the gut microbiota associated with CKD progression. We also combined correlation analysis to identify clinical features closely related to the gut microbiome. Our results offered the possibility of using non-invasive gut microbiome in the early diagnosis of course from CKD to ESRD and provide new insights into the association between clinical features and gut microbiota in CKD.

## Introduction

CKD is a disease of decreased renal function due to structural and functional alterations of renal, and is usually diagnosed clinically by estimated glomerular filtration rate (eGFR) below 60 mL/min/1.73m^2^ for at least three months (Wang et al., 2019; Collaborators, G. B. D. R. F, 2020). According to incomplete statistics, approximately 15-20% of adults worldwide suffer from CKD, and by 2040, CKD will be the fifth leading cause of death all over the world (Chronic Kidney Disease Prognosis, C et al., 2010; Foreman et al., 2018). The occurrence of CKD leads to some complications such as progressive uremia, anemia, volume overload, electrolyte abnormalities, mineral, and bone disorders, which in turn have severe and widespread impacts on the cardiovascular system. Consequently, cardiovascular complications of CKD are a major cause of death in patients (Matsushita et al., 2022). These complications have also allowed CKD to progress from kidney damage with normal renal function to ESRD with high mortality (Zarantonello et al., 2021). Patients with ESRD have substantial loss of kidney function and can essentially only be sustained with renal transplantation or dialysis (Kalantar-Zadeh et al., 2021). Therefore, early attention to these indicators of complications in patients with CKD has a positive preventive effect in avoiding the development of ESRD.

The intestine is an important organ for digestion and absorption, and has a crucial impact on human metabolism. Gut and the colonized microbiome constitute the human intestinal microecosystem, which is essential for human health (Dalton et al., 2019). Recently, the introduction of the concept of the gut-kidney axis has shed new light on the mechanism of the bidirectional interaction between the intestine and the kidney (Sato et al., 2021). As a result, the role of gut microbiota in CKD is receving increasing attention. As the pathogenic stage of CKD progresses to ESRD, the concentration of urea accumulates gradually in the blood (Chi et al., 2021), and the intestine becomes the main route of urea excretion due to the decrease or even loss of metabolic function in the kidney. The massive influx of urea into the gastrointestinal tract and the secretion of uric acid and oxalate by the colonic epithelium (Simoes-Silva et al., 2018), create a disrupted intestinal epithelial barrier and increase intestinal permeability, profoundly altering the biochemical environment of the intestine (Hatch and Vaziri, 1994; Vaziri et al., 1995). Alterations of intestinal microenvironment also led to changes in the homeostasis and composition of the gut microbiota. Studies have shown that the Actinobacteria, Firmicutes and Proteobacteria are significantly increased, while the *Lactobacillaceae* and *Prevotellaceae* are greatly decreased in CKD patients (Vaziri et al., 2013), and a large number of bacteria with urease, uricase, indole and p-cresol forming enzymes were observed in ESRD patients (Wong et al., 2014). Meanwhile, metabolites of gut microbes also contribute to the progression from CKD to ESRD and the generation of complications. The severely abnormal gut microbiota in ESRD patients has been shown to have the functional potential to accelerate the biosynthesis of many toxic compounds (Wang et al., 2020). Gut microbes are responsible for synthesizing toxins that can accumulate in the patient’s bloodstream and resist removal by dialysis (Aronov et al., 2011; Tanaka et al., 2015). Consequently, it is crucial to address alterations in gut microbial homeostasis in CKD patients to prevent the occurrence of CKD and delay disease progression.

In conclusion, complications caused by CKD may advance the progression of CKD to ESRD, and the gut microbial community of individuals with CKD can be significantly affected and exacerbated bidirectionally by the interaction of the gut-kidney axis. Several researches have involved the relationship between clinical indicators of patients with CKD and their gut microbial dynamics, but a comprehensive analysis is still required. Only alterations in the genera *Parasutterella*, *Lactobacillus*, *Paraprevotella*, *Clostridium sensu stricto*, and *Desulfovibrio* have been reported to be associated with CKD disease severity indicators including eGFR, and *Akkermansia* was significantly inversely correlated with production of interleukin-10 (Li et al., 2019). In this study, we performed high-throughput sequencing of feces from patients with CKD (including ESRD) combined with various bioinformatics analyses, expecting to illustrate the steady-state changes in gut microbial communities of patients with CKD and ESRD. We also intended to explore the relationship between clinical indicators and gut microbial communities, with the aim of providing a reference for clinical diagnosis based on changes in microbial communities.

## Materials and methods

### Sample collection

We recruited participants into our study by questioning among patients admitted to the Third Xiangya Hospital (Hunan Province, China) with a confirmed ESRD or CKD hospitalization during February-March 2022. We selected patients aged 22-76 years and collected a total of 16 stool samples (11 males and 5 females). We provided all patients with detailed information about our study, including the purpose, procedure, potential risks and benefits. And all patients signed an informed consent form. We also ensured that all patient information was kept strictly confidential and anonymous. Those using laxatives, probiotics, antidiarrhoeal drugs, drugs for the treatment of rheumatoid arthritis (RA), glucocorticoids, immunosuppressive agents, antineoplasm agents and antimicrobials were excluded from the studies. Finally, we selected only 13 cases for inclusion in our study. The study was approved and registered by the Institutional Review Board of Third Xiangya Hospital, Central South University (2021-S000). Clinical and demographic information was recorded for each patient, including age, gender, dialysis duration, and basic inflammatory information. All stool samples collected were first stored at -80°C, labeled, and then for DNA extraction and sequencing simultaneously.

### DNA extraction, 16S rRNA gene sequencing and data processing

Total genomic DNA samples were extracted using the OMEGA Soil DNA Kit (M563 6-02) (Omega Tek, Norcross, GA, USA) following the manufacturer’s instructions, and stored at -20°C prior to further analysis. The quantity and quality of extracted DNAs were measured using a NanoDrop NC2000 spectrophotometer (Thermo Fisher Scientific, Waltham, MA, USA) and agarose gel electrophoresis, respectively.

PCR amplification of the V3–V4 region of bacterial 16S rRNA genes was performed using the forward primer 341F (5’- CCTAYGGGRBGCASCAG-3’) and the reverse primer 806R (5’-GGACTACNNGGGTATCTAAT-3’). Thermal cycling consisted of an initial denaturation at 98°C for 5 min, followed by 25 cycles consisting of denaturation at 98°C for 30 s, annealing at 53°C for 30 s, and extension at 72°C for 45 s, with a final extension of 5 min at 72°C. PCR amplicons were purified with Vazyme VAHTSTM DNA Clean Beads (Vazyme, Nanjing, China) and quantified using the Quant-iT PicoGreen dsDNA Assay Kit (Invitrogen, Carlsbad, CA, USA). Sample DNA library and pair-end 2×250 bp sequencing was performed using the Illlumina NovaSeq platform with NovaSeq 6000 SP Reagent Kit (500 cycles) at Shanghai Personal Biotechnology Co., Ltd (Shanghai, China).

Briefly, raw sequence data were demultiplexed using the demux plugin following by primers cutting with cutadapt plugin (Martin, 2011). Sequences were then quality filtered, denoised, merged and chimera removed using the DADA2 plugin (Callahan et al., 2016). Sequences were clustered at the 97% similarity level (default) using Vsearch (Rognes et al., 2016). Qiime2 (version 2021.11) was employed to calculate beta diversity (Bolyen et al., 2019). Using Silva (Release 132) (Quast et al., 2012) as the reference database, the feature sequences was annotated by Bayes classifier, and the species classification information corresponding to each feature was obtained. Then, the community composition of each sample was counted at different levels and the species relative abundance tables of species at different taxonomic levels were generated by using QIIME2 (version 2021.11) software (Bolyen et al., 2019).

### Statistical analysis

**Figure 1:**
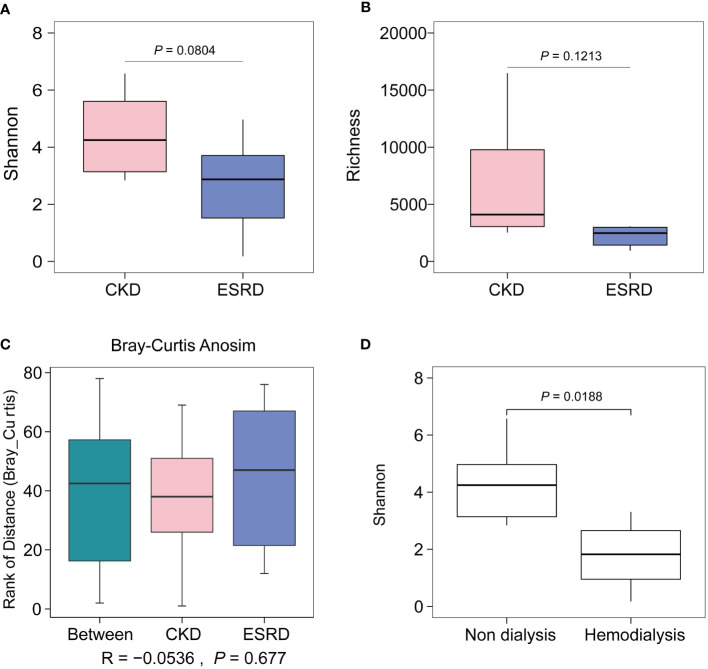
Alpha and beta diversity of CKD and ESRD group. **(A)** Shannon index, **(B)** Richness index shown the alpha diversity between two groups. **(C)** Comparison of significance of beta diversity between CKD and ESRD groups. **(D)** Comparison of Shannon Wiener according to whether hemodialysis or not. Data were analyzed by t.test.

All statistical analyses were performed using the R V4.1.2 environment (R Core Team, 2022). In the absence of special instructions, the statistical results were visualized using the “ggplot2” package (Wickham, 2016). Baselines were generated using the “tableone R” package (Yoshida and Bartel, 2022). Alpha diversity was measured using the function diversity in the package “vegan” based on flattening taxonomy table (Oksanen et al., 2022). Beta diversity was compared using Principal Coordinate Analysis (PCoA), which compares bacterial community composition across all samples based on Bray-Curtis distances. Beta diversity between sample groups was compared by PERMANOVA with 999 permutations, ANOSIM was selected to test for significance between groups (Wang et al., 2008), R > 0, *P* < 0.05 was considered significant. A phylogenetic tree of the abundant OTUs (>= 0.2%) was constructed by the iTOL online tool (Letunic and Bork, 2021). The Venn plot was generated by “VennDiagram” R package (Chen, 2022). The “linkET” package was used for the mantel test plot (Huang, 2021). The “DEseq2” package was used to analyze the relative abundance difference and marker genera (Topper et al., 2017). The differential expression matrix and the *P*-value (adjusted) matrix of species composition were obtained through the function DESeqDataSetFromMatrix. The significance level was *P*_adj_ < 0.05 and absolute FoldChange value was greater than 1. The “vegan” package was used to calculate the Spearman association analysis of environmental factors with the composition of the top 50 abundant genera, and the heatmap was drawn with the “pheatmap” package (Kolde, 2019; Oksanen et al., 2022). The *P*-value from **Figures 1**, **S5** were corrected by the Benjamini and Hochberg multiple test to control the false discovery rate (FDR) (Benjamini and Hochberg, 1995). The “vegan” package was also employed to conduct Canonical Correspondence Analysis (CCA) and envfit test (Oksanen et al., 2022). The network showed interaction between genera and clinical indicators were established based on Spearman correlation analysis by the “igraph” package and Gephi software (version 0.10.0) (Csardi and Nepusz, 2006; Bastian et al., 2009). We set the threshold for the *P*-value corrected by the Benjamin and Hochberg FDR to 0.05.

## Results

### Clinical information of patients

A total of 16 fecal samples were collected prospectively from different patients. After rigorous diagnosis and exclusion procedures, 13 fecal samples were included for analysis, including 7 samples in the CKD group and 6 samples in the ESRD group. We characterized the gut microbiome of patients in these 2 groups by 16S rRNA sequencing.

In both CKD and ESRD groups, patients were matched for gender, age, and clinical indicators (**Table 1**). There were significant differences in the eGFR, creatinine (Cr), blood urea nitrogen (BUN), Lymphocytes (L) and carbon dioxide (CO_2_) between the two groups **(** **Table 1** **)**. eGFR, Cr and BUN were used as indicators to evaluate renal function (Wuttke et al., 2019; Kashani et al., 2020; Kjaergaard et al., 2022). Lymphocytes was an indicator to assess the presence of a bacterial or viral infection (Cao et al., 2021). CO_2_ was measured during renal function tests to assess for the existence of metabolic alkalosis (Cao et al., 2021).

**Table 1:** Clinical indicators of patients in the CKD and ESRD group.

	Overall (*n* =13)	CKD (*n* =7)	ESRD (*n* = 6)	*P-*value
Gender (Female/Male)	3/10	2/5	1/5	
Age (year)	55.538 ± 10.170	53.286 ± 12.867	58.167 ± 5.845	0.412
Dialysis (0/1)	9/4	7/0	2/4	0.0462
eGFR	21.662 ± 20.063	36.547 ± 15.015	4.296 ± 4.844	0.0004
Cr [μmol L^-1^]	547.692 ± 388.437	273.286 ± 216.984	867.833 ± 277.877	0.0012
Hb [g L^−1^]	102.000 ± 30.042	109.000 ± 33.541	93.833 ± 25.833	0.3873
WBC [×10^9^ per L]	8.098 ± 3.664	9.803 ± 3.034	6.108 ± 3.516	0.0666
HCT %	31.062 ± 8.707	32.614 ± 9.789	29.250 ± 7.724	0.5113
PLT [×10^9^ per L]	209.231 ± 78.309	240.571 ± 87.831	172.667 ± 49.810	0.1229
L %	1.605 ± 0.915	2.227 ± 0.769	0.880 ± 0.357	0.0024
N [×10^9^ per L]	5.678 ± 2.683	6.720 ± 2.391	4.463 ± 2.670	0.136
TP [g L^−1^]	60.800 ± 56.600, 62.600	60.800 ± 50.350, 62.950	60.700 ± 57.500, 62.175	0.8864 (nonnorm)
ALB [g L^−1^]	31.062 ± 7.245	28.814 ± 8.433)	33.683 ± 5.033	0.2429
BUN [mg dL^-1^]	19.317 ± 10.015	12.754 ± 5.807	26.973 ± 8.344	0.0041
UA [μmol L^-1^]	464.300 ± 184.127	526.857 ± 132.546	391.317 ± 220.032	0.1978
CO_2_ [mmol L^-1^]	21.900 ± 18.200, 24.100	24.100 ± 22.600, 24.950	19.100 ± 17.675, 20.825	0.0455 (nonnorm)
LDL_C [mmol L^-1^]	2.421 ± 1.510)	3.141 ± 1.701)	1.580 ± 0.651)	0.0586
HDL_C [mmol L^-1^]	0.950 ± 0.830, 1.120	0.950 ± 0.885, 1.130	0.905 ± 0.723, 1.080	0.5197 (nonnorm)
K [mmol L^-1^]	4.145 ± 0.650	4.003 ± 0.401	4.310 ± 0.873	0.4197
Na [mmol L^-1^]	139.615 ± 2.347	140.343 ± 1.876	138.767 ± 2.718	0.2432
Cl [mmol L^-1^]	102.908 ± 3.521	104.843 ± 3.284	100.650 ± 2.336	0.0244
Ca [mmol L^-1^]	2.122 ± 0.130	2.139 ± 0.148	2.103 ± 0.116	0.6463
P [mmol L^-1^]	1.509 ± 0.501)	1.336 ± 0.297	1.712 ± 0.637	0.1885
Mg [mmol L^-1^]	0.924 ± 0.137)	0.863 ± 0.093	0.995 ± 0.152	0.0805
Tchol [mmol L^-1^]	4.160 ± 2.870, 4.870	4.310 ± 4.215, 6.400	2.665 ± 2.407, 3.575	0.0633 (nonnorm)
TG [mmol L^-1^]	2.010 ± 1.750, 2.250	2.210 ± 2.005, 2.480	1.435 ± 1.030, 1.960	0.1161 (nonnorm)
PTH [pg mL^-1^]	307.113 ± 272.039)	240.572 ± 242.530	351.473 ± 303.336	0.5594
Ferritin [ng mL^-1^]	647.692 ± 478.444)	570.714 ± 385.515	737.500 ± 594.189	0.5543
TRF [g L^-1^]	1.552 ± 0.410)	1.531 ± 0.426	1.575 ± 0.43	0.8581
β2-MG [mg L^-1^]	25.199 ± 9.586)	16.954 ± 2.188	34.818 ± 2.928	<0.0001
CRP [mg L^-1^]	5.630 ± 3.900, 63.600	5.315 ± 3.950, 20.123	51.420 ± 3.900, 135.600	0.4624 (nonnorm)
PCT [ng mL^-1^]	3.477 ± 8.675)	0.062 ± 0.040	8.029 ± 13.090	0.2633
BNP [ng L^-1^]	5278.948 ± 7077.542	855.757 ± 903.042	9702.138 ± 7993.287	0.0702
VD [ng mL^-1^]	8.346 ± 7.698	2.158 ± 2.236	12.987 ± 6.941	0.0512

CKD, chronic kidney disease; eGFR, estimated glomerular filtration rate; Cr, serum creatinine; Hb, hemoglobin; WBC, white blood cell count; HCT, hematocrit; PLT, platelet; TP, treponema pallidum; ALB, albumin; BUN, blood urea nitrogen; UA, uric acid; LDL_C, low-density lipoprotein cholesterol; HDL_C, high-density lipoprotein cholesterol; Tchol, total cholesterol; TG, triglyceride; PTH, parathyroid hormone; TRF, transferrin; β2_MG, β2 microglobulin; CRP, C-reactive protein; PCT, procalcitonin; BNP, B-type natriuretic peptides; VD, vitamin D. Continuous variables were expressed as means ± standard deviations, while nonnormal continous variables were expressed as median ± interquartile ranges. Continuous variables were compared using Oneway test, and categorical variables were compared using Chisq test, Kruskal test was used for the nonnormal continous variables. (The “0” and “1” in the dialysis unit indicate whether the patient is on dialysis or not, respectively).

### The diversity of gut microbiome in cohort

After data cleaning and clustering of all fecal sample sequencing data, a total of 50,727 OTUs were detected. The Shannon Wiener curve represents that the sequencing depth is sufficient to perform the subsequent analyses **(** **Figure S1** **)**. As the result of calculating the indices of alpha diversity of OTUs, Shannon index **(** **Figure 1A** **)**, Richness index **(** **Figure 1B** **)** decreased with the disease progression without any significant difference. The PCoA and ANOSIM test were employed in beta diversity comparison, the observations did not produce any significant clustering **(** **Figures 1C**, **S2** **)**. Besides, some differences were also observed in the gut microbial indices of alpha diversity depending on whether hemodialysis was performed or not **(** **Figure 1D** **)**.

### More variation of genus community composition in CKD

**Figure 2:**
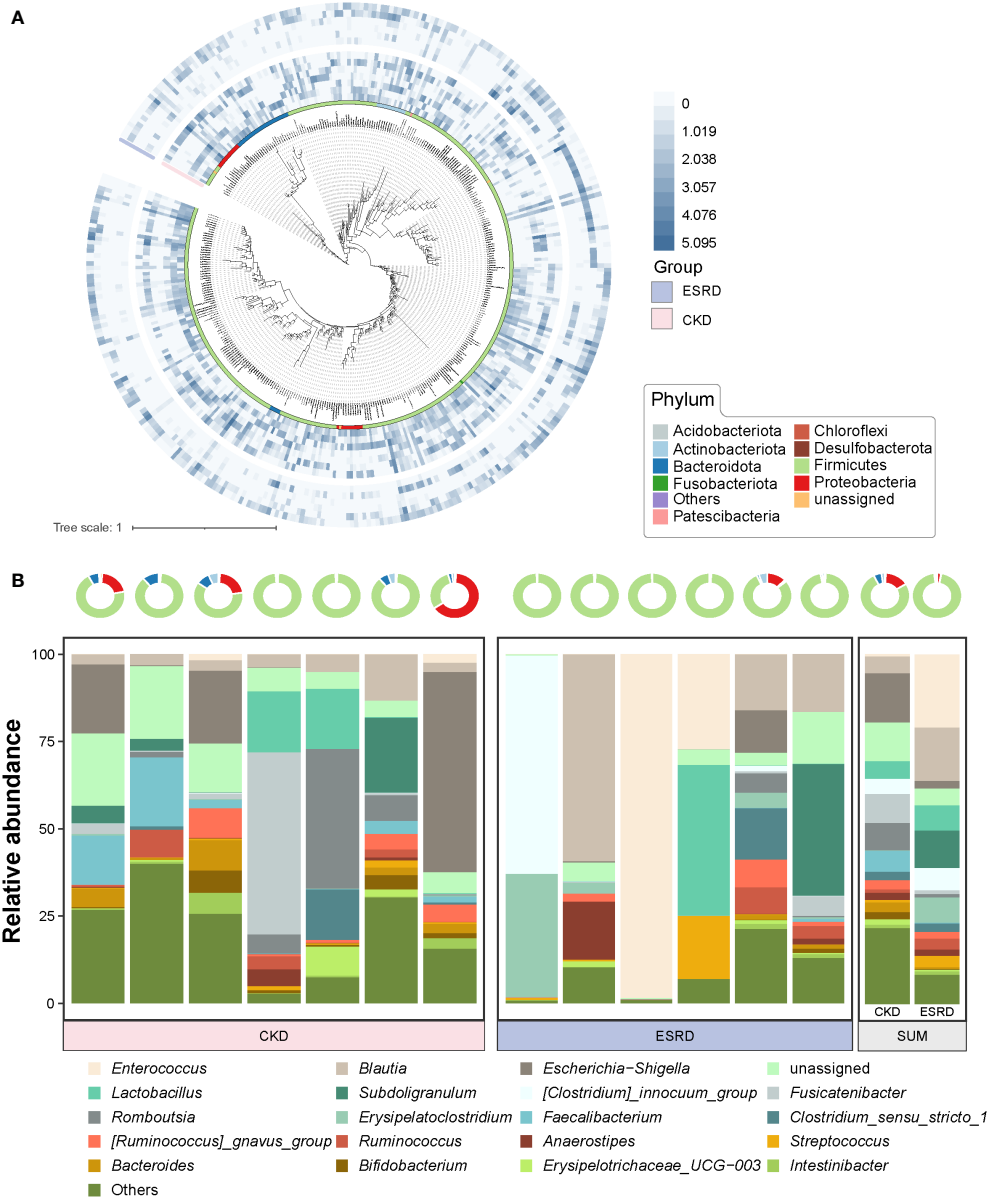
Evolutionary relationships and composition of gut microbiota in the CKD and ESRD groups **(A)** Circular maximum likelihood phylogenetic tree of high relative abundance of OTUs (> 0.02%). The inner band shows OTUs colored by phylum. The out heatmap present the relative abundance of OTUs in each sample. **(B)** Ring plot represented the relative abundance in top 10 phylum of all samples. Stacked bar plot represents the relative abundance of top 20 genera in all samples.

**Figure 3:**
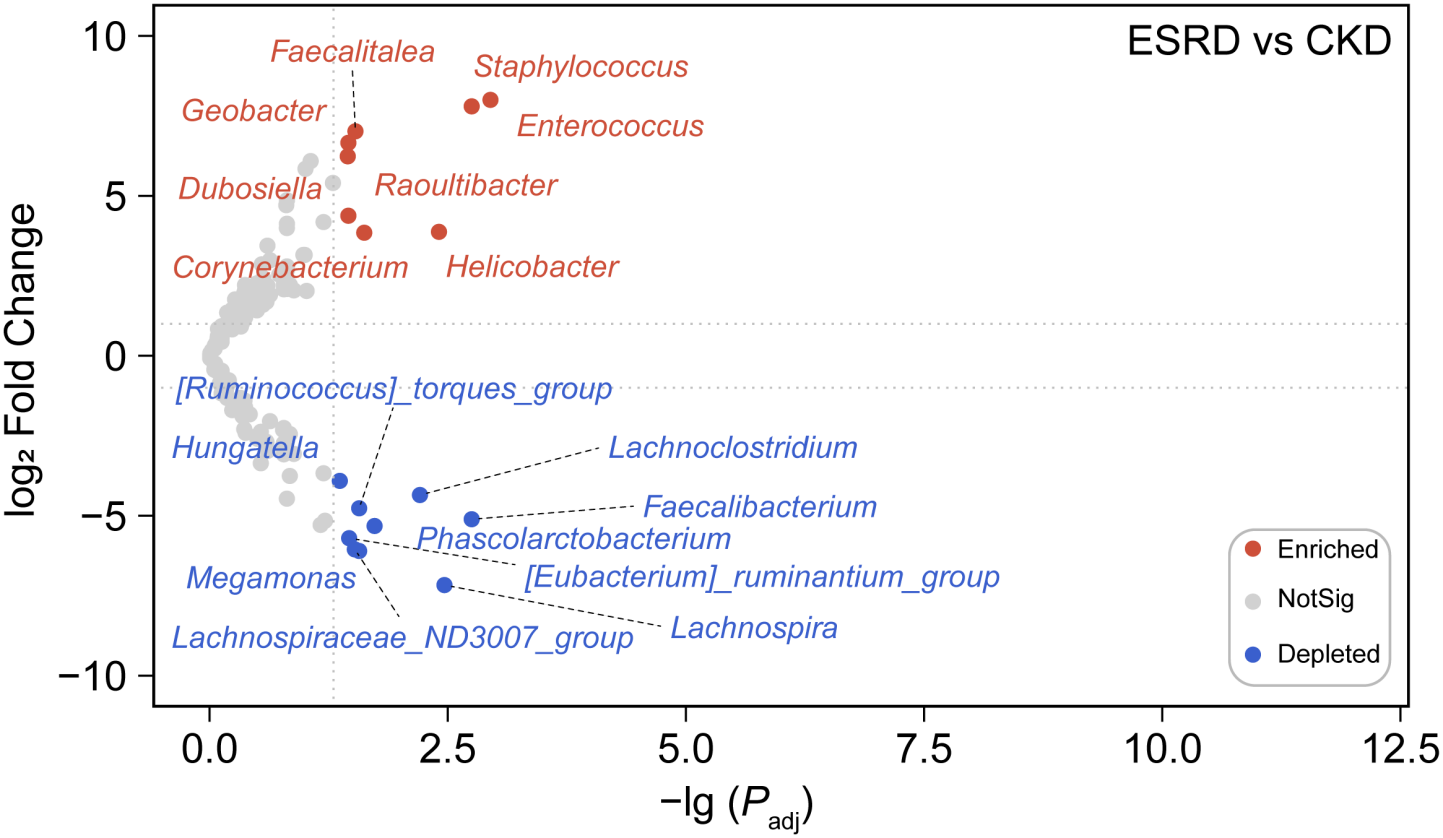
Differential abundance analysis at the genus level. The volcano revealed a marked difference in the abundance of certain genera between the ESRD group and the CKD group. The nodes in the plot were color-coded to reflect the type of change observed, blue dots indicate depleted genera, red dots indicate enriched genera, and gray dots indicate no significant difference between groups.

We screened OTUs with higher relative abundance (>0.02%) to construct a phylogenetic tree **(** **Figure 2A** **)**. The difference of relative abundance of OTUs between CKD and ESRD groups was indicated by heatmaps in the outer circles **(** **Figure 2A** **)**. Interestingly, there was a significant difference in the relative abundance of the filtered OTUs between CKD and ESRD groups **(** **Figure S3** **)**. Moreover, in the Circular maximum likelihood phylogenetic tree and ring plot, Firmicutes had the highest number of compositions and relative abundance, followed by Proteobacteria, Bacteroidota and Actinobacteriota at the phylum level **(** **Figures 2A, B** **)**. From the Venn plot at the genus level, there were more members of the genus community in the CKD group than in the ESRD group **(** **Figure S4** **)**. *Escherichia-Shigella, Blautia, Fusicatenibacter, Romboutsia* and *Faecalibaterium* had the massive relative abundance in the CKD group, while the *Enterococcus*, *Blautia, Subdoligranulum* and *Lactobacillus* were dominant in the ESRD group **(** **Figure 2B** **)**. However, the results presented a large variation between samples, the differences within groups appeared to be greater than the differences between groups. There were some samples where a single genus accounted for more than 50% of the relative abundance, suggesting a simplification of the patient’s gut microbiota with the development of CKD. Compared to the CKD group, we observed that 8 genera (marked in red) were significantly increased, while 9 (marked in blue) were significantly declined in the ESRD group **(** **Figure 3** **)**. *Faecalitalea*, *Enterococcus*, *Staphylococcus*, *Geobacter*, *Dubosiella*, *Raoultibacter*, *Helicobacter*, and *Corynebacterium* were significantly increased, while *Lachnoclostridium*, *Faecalibacterium*, *Phascolarctobacterium*, *Lachnospira*, *Megamonas*, *Hungatella*, *Lachnospiraceae_ND3007_group*, *[Ruminococcus]_torques_group*, and *[Eubacterium]_ruminantium_group* were significantly decreased in the ESRD group.

### Relationship between gut microbial communities and clinical indicators in CKD patient’s cohort

Clinical indicators are closely related to the diagnosis of kidney disease. We further uncovered the association of the concerned clinical indicators with the composition of the intestinal microbiota. We first calculated the correlation between clinical indicators and the top 50 genera in both groups by Spearman analysis method and plotted the heatmap **(** **Figure S5** **)**. Apparently, there were more clinical indicators significantly associated with abundant genera in the CKD group than in the ESRD group (* *P*-value <0.05, ** *P*-value < 0.01). The VD and BNP indicators were significantly associated with some of the top 50 genera in both groups. In addition, UA, HDL_C, TG, and β2_MG were also significantly associated with some of the top 50 genera in the ESRD group **(** **Figure S5** **)**.

**Figure 4:**
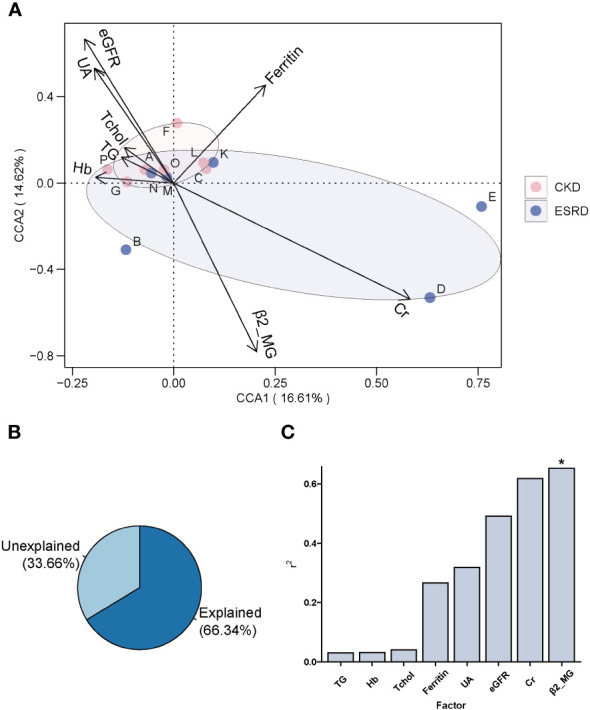
The association between clinical indicators and genera composition. **(A)** Clinical indicators explanation for changes in the gut microbiota of patients by CCA analysis. **(B)** The amount of variation in community structure explained by pathogenetic factors. **(C)** Histogram of the amount of variation in community structure explained by each pathogenetic factor. (envfit analyze, *P-value < 0.05).

The CCA analysis of the CKD and ESRD groups gut microbiome and these clinical indicators is shown in **Figure 4A**. All these clinical indicators possessed 66.34% explanatory degree of the community structural changes **(** **Figure 4B** **)**. Notably, the composition of the patients’ gut microbial community of patients was mainly influenced by β2_MG (envfit analyze, * *P*-value < 0.05) (**Figure 4C**).

### Correlation between clinical indicators and genera with different abundance

**Figure 5:**
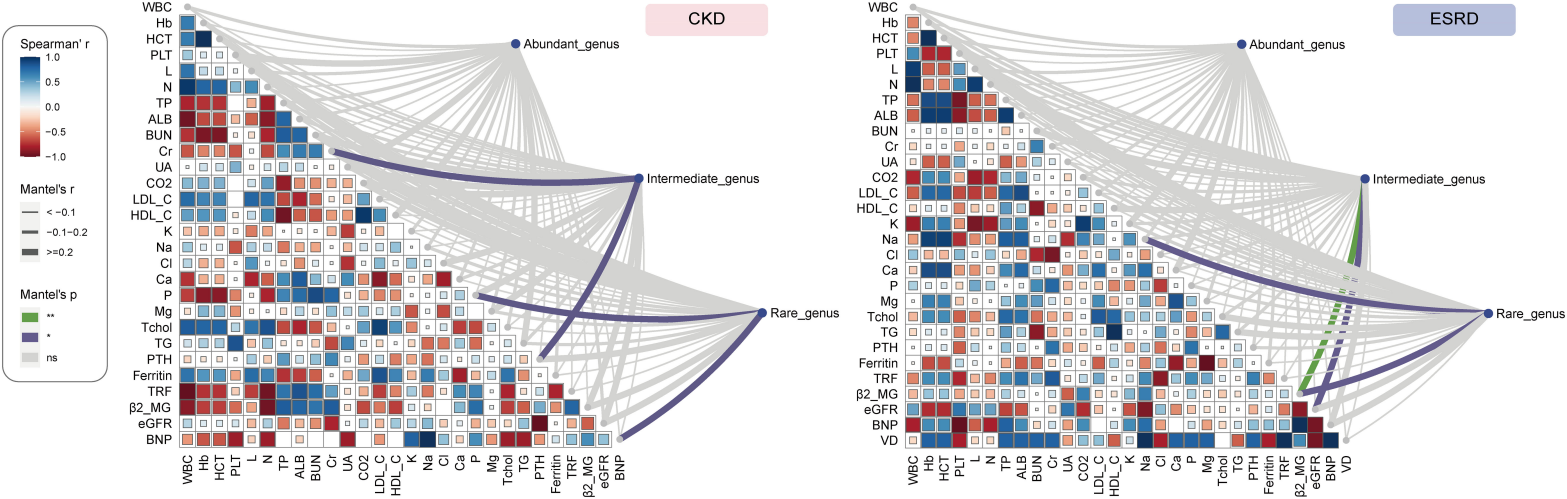
The Association of clinical indicators with genera abundance. Mantel test show the relationship between of all the abundant genus, intermediate genus, rare genus abundance and clinical indicators in CKD and ESRD group.

To continue investigate the relationship between gut microbial composition and clinical indicators, we divided the genera into 3 groups according to their relative abundance: abundant genus (> 0.2%), intermediate genus (0.2% - 0.01%) and rare genus (< 0.01%). The Mantel test can be performed to test the relationships between clinical indicators and gut microbial structure, so it was employed to analyze the potential correlation between clinical indicators and different genus group on both CKD and ESRD groups. Obviously, the intermediate genera were significantly positively associated with Cr and PTH, while rare genera were significantly positively correlated with P and BNP in CKD group **(** **Figures 5**, **S6A–D** **)**. On the other hand, the clinical indicator β2_MG was significantly positively associated with intermediate and rare genera, the Na and eGFR were significantly positively related to intermediate and rare genera respectively **(** **Figures 5**, **S6E–H** **)**. All the results above indicated that not only the composition of abundant genera in the intestine was influenced by clinical indicators, but also the intermediate and rare genera. We also performed the analysis of network about the correlation between clinical indicators and genera with significant difference in relative abundance. Based on the results, we found that *Phascolarctobacterium* had a significant negative correlation with β2_MG, while *Raoultibacter* showed the opposite correlation with eGFR. In addition, *Faecalitalea* presented a positive correlation with Cr and a negative correlation with Cl. The above results were only shown in the ESRD group, and cannot be found in the CKD group **(** **Figure S7** **)**.

## Discussion

Maintaining the homeostasis of the gut microbial community plays an important role in human health. With the progressive maturation and development of high throughput sequencing technologies, the role of gut microbiome homeostasis in various types of chronic diseases has become increasingly prominent. Disruption of the homeostasis of the gut microbial environment has been proven to be associated with accelerated disease progression and progressive deterioration in various types of chronic diseases, such as diabetes (Qin et al., 2012), and obesity (Ley et al., 2006). In recent years, the relationship between CKD and the gut microbiota has been increasingly reported, and studies have shown that changes in the gut microbiota are significantly related to the disease process of CKD (Sampaio-Maia et al., 2016). Several studies have demonstrated that there are potential relationship between clinical indicators and feature microbe in the gut of CKD patients, but systematic analyses and descriptions were lacking. In this study, we explored the changes of gut microbial community homeostasis during the disease course of CKD by 16S rRNA sequencing technology combined with various bioinformatics analysis. To explore the indication and representative role of gut microbes during the evolution of CKD, correlation analysis between gut microbial communities and clinical indications was also employed. We collected fecal samples from clinical patients with CKD and observed the changes in the diversity, composition of intestinal microbial colonies during the evolution of CKD into ESRD, and analyzed the intestinal microbes associated with them in combination with clinical characteristics indicators.

During the progression from CKD to ESRD, we observed no significant difference in the alpha diversity and beta diversity of the gut microbiome, but a trend toward a decrease in alpha diversity. Consistent with previous studies, alpha diversity was reported to show a decreasing trend but also did not reach significance in the middle and end stages of CKD disease (Jiang et al., 2017). The lack of significant difference in diversity indices between samples in our cohort is likely due to the fact that all patients were in the middle or end stages of CKD and the patient information was biased towards aging. However, a significant difference in alpha diversity was observed between ESRD and non-CKD patients, indicating that the imbalance of the intestinal microbiome is associated with the pathogenic process of CKD (Sampaio-Maia et al., 2016). With the aggravation of the disease and the accumulation of the onset time, the gut microbiota of patients with ESRD would be disordered to a greater extent than that of healthy people (Luo et al., 2021). However, the relative abundance and diversity of the intestinal microbiota will decrease in middle and end stage of CKD patients as the condition worsens, the gut microbial composition gradually stabilizes and the degree of dysbiosis no longer fluctuates. Interestingly, we observed that dialysis treatment in CKD patients significantly affects the diversity of the gut microbial community, which is also consistent with the study of Luo (Luo et al., 2021). In summary, the progression of CKD affects the diversity of the gut microbiome, but the imbalance of the intestinal microbiota is alleviated and the gut microenvironment is stabilized in the middle and end stages of CKD.

According to the previous studies, the composition of intestinal microbial colonies in the ESRD and CKD patients also showed a trend toward of simplification compared with the healthy human body (Ren et al., 2020; Luo et al., 2021). By analyzing the composition of the intestinal microenvironment in patients with CKD, we found that the intestinal microbial community was significantly changed with the condition of CKD deteriorates. And the relative abundance of high abundance OTUs (relative abundance > 0.02%) in ESRD group were significantly decreased compared to CKD group. In addition, the community composition at phylum level and genus level tended to become simpler gradually. At the phylum level, the gut microbiota of patients with CKD was mainly dominated by Firmicutes, and the relative abundances of other phyla were all significantly declined during the progression of kidney disease to ESRD, but Firmicutes dominated. Patients with CKD had a significant reduction in the compositional complexity at the genus level and a trend toward a simplistic community structure, suggesting that disease exacerbation in CKD leads to a change in the gut microenvironment and thus a trend toward a monomorphic gut microbial community. Through differential abundance analysis at the genus level, we observed a remarkable increase in the relative abundance of *Staphylococcus*, *Enterococcus* and *Helicobacter*, indicating that these intestinal microorganisms play an important role in the disease progression of CKD. Meanwhile, we also found a decrease in *Faecalibacterium* and *Lachnospira*, which was similar to the previous studies (Forbes et al., 2018; Zhao et al., 2021). *Faecalibacterium* is a bacterium that is capable of producing butyrate. Then, Butyrate is an anti-inflammatory compound that exerts nephroprotective effects by inhibiting the expression of histone deacetylases, thus reducing fibrosis and attenuating acute kidney injury (Brilli et al., 2013; Fujio-Vejar et al., 2017; Felizardo et al., 2019). Hence, the reduction in the relative abundance of *Faecalibacterium* led to a decrease butyrate production, which may be one of the explanations for the deterioration of CKD. Furthermore, *Lachnospira* has been suggested to play a protective role in inflammation from a study(Wright et al., 2017). In addition, there has not been much research on these genera such as *Geobacter* and *Raoultibacter* in the study of kidney disease and intestinal microbiota, which means that they may be new indicator genera in the exacerbations of CKD. As a result, we are able to provide a reference for elucidating the mechanism underlying the relevance of the gut microbiota and the pathological process of CKD in terms of the different relative abundance of the genus and the composition of the gut microbiota.

Accumulating evidence has demonstrated the important impact of metabolites from the gut microbiome on immune function and metabolic diseases (Zhernakova et al., 2016). Not only can the gut microbiome produce beneficial metabolites such as short chain fatty acids (Sadler et al., 2020), but also it can also produce harmful substances that promote the disease processes. In the related studies of CKD disease, uremic toxins derived gut were confirmed to promote the disease process of CKD and the prevalence probability of its complications (Meijers et al., 2010; Wang et al., 2020). Interestingly, we found that β2-MG was an important factor affecting the composition of the intestinal microbial community. In the analysis of CCA and Mantel test of the correlation between community structure and clinical indicators, we found that β2_MG have a significant effect on the structure of gut microbial communities. Most of the clinical indicators that showed significant correlation were mainly related to the intermediate and rare genus. And all of them showed positive correlation. Moreover, the bacterial groups of intermediate and rare abundance showed a significant positive correlation in the ESRD phase, indicating that an increase in β2-MG can promote the growth of a specific group of gut microbiota. β2-MG is a class of endogenous proteins that have been shown in numerous studies to cause relevant amyloidosis in patients with ESRD, leading to bone and joint degeneration and even loss of musculoskeletal system function (Winchester et al., 2003; Corlin and Heegaard, 2012; Musial and Zwolinska, 2019), increasing the probability of musculoskeletal-related complications in CKD. However, studies on the association of β2_MG with intestinal microbiota are largely absent. In addition, clinical indicators of CKD complications, such as abnormal blood electrolytes, uremia, and cardiovascular disease, were shown to be significantly and positively correlated with rare and intermediate abundance genera structure, suggesting an important role of intermediate and rare abundance genera in revealing ESRD-related clinical indicators. Meanwhile, we also found significant correlation between clinical indicators and genus with significant difference in relative abundance based on the network analysis. *Phascolarctobacterium* showed the significant negative correlation with β2_MG in the ESRD patient. Similar result was also discovered in the Mental analysis that the β2_MG maybe influence the composition of gut microbiome. Furthermore, *Phascolarctobacterium* was a potentially beneficial bacterium that could be involved in metabolic regulation of the host by producing SCFAs (Li et al., 2022). And it was rich in the gut of healthy people compared to the patients (Wang et al., 2021). The negative correlation of β2_MG with *Phascolarctobacterium* provided new insights for the association analysis between clinical indicators of other diseases and different species. In conclusion, we demonstrated the correlation between relevant clinical indicators and the structure of gut microbiota in patients with middle and advanced stage CKD, which may provide an important direction for further exploration of intestinal microbiota-based methods for the early clinical diagnosis of CKD.

In this study, we identified an important role of the gut microbiota and its metabolites in the disease course of CKD. After reviewing the experiments, we still need some improvements in the following. First, we acknowledge that this study requires second validation cohort, and the larger sample size. More samples lead to more plausible results. However, after rigorous analysis and multiple tests of the data, such as the Benjamini and Hochberg multiple test, the results obtained from the analysis of the samples involved in this study are authentic and credible (Benjamini and Hochberg, 1995). Furthermore, based on some studies, we found our results are in agreement with previous studies, such as the same diversity variation trend and similar differential genera (Jiang et al., 2017; Forbes et al., 2018; Zhao et al., 2021). These results also contribute to the credibility of our findings. Second, long-term follow-up of patients will be able to better interpret the dynamic changes in the gut microbiota during CKD and reduce the errors caused by individual differences. Then, since the metabolites produced by gut microbes can play an important role in the CKD disease process, the introduction of gut microbial metabolomics and CKD correlation studies may make the results more convincing. In conclusion, further studies are needed to explain the mechanisms of effect of gut microbial alterations in the gut and correlations with clinical indicators in patients with middle and end stages of CKD. Our results could bring more possibilities for kidney disease diagnosis and remission in the future.

## Data availability statement

The repositories and accession numbers can be found below: https://ngdc.cncb.ac.cn/gsa; CRA008974. The datasets presented in this study can be found in the Genome Sequence Archive online repositories (Chen et al., 2021) in National Genomics Data Center (CNCB-NGDC Members and Partners et al., 2022), China National Center for Bioinformation / Beijing Institute of Genomics, Chinese Academy of Sciences.

## Ethics statement

The studies involving human participants were reviewed and approved by IRB of Third Xiangya Hospital, Central South University (2021-S000). The patients/participants provided their written informed consent to participate in this study.

## Author contributions

JWW and JH conceived the study. QO and XP collected the fecal sample form CKD patients. JYW, HC and ZY analyzed the data, wrote, and edited its final manuscript. All authors contributed to the article and approved the submitted version.
