# Supplementary material for: Alterations of gut microbes and their correlation with clinical features in middle and end-stages chronic kidney disease
Source: Front Cell Infect Microbiol. 2023 Mar 24;13:1105366. doi: 10.3389/fcimb.2023.1105366 (PMC10079997; doi:10.3389/fcimb.2023.1105366)
Supplement: Supplementary file 1 [file DataSheet_1.docx]

Supplementary Material

## Supplementary Figures


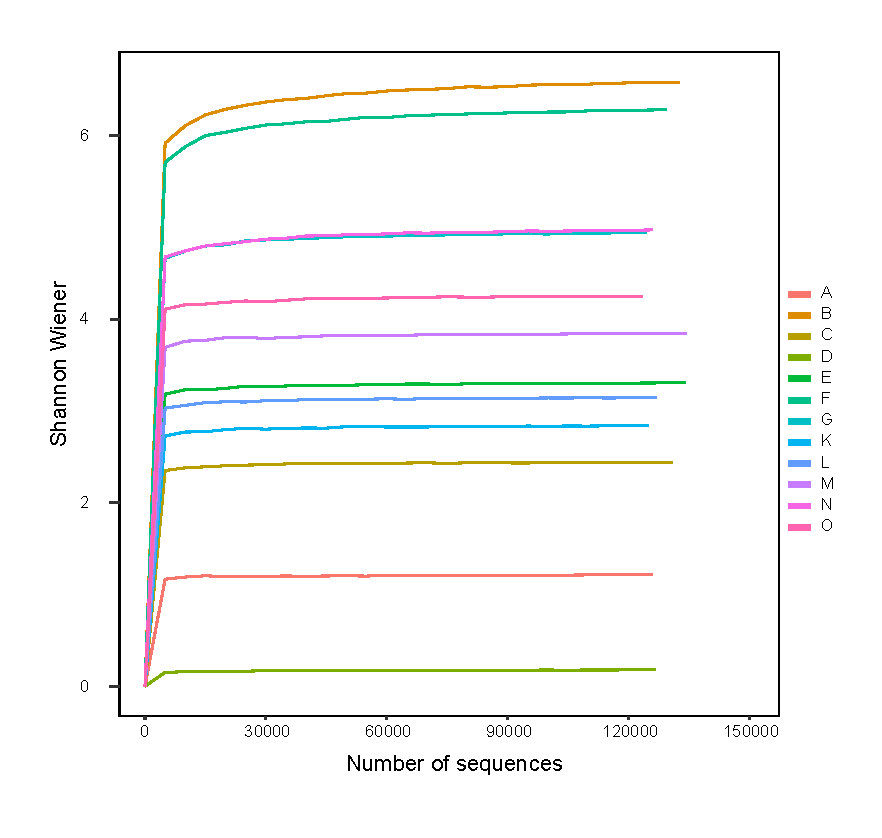


**Supplementary Figure 1**. Alpha diversity Shannon Wiener curve of all sample.


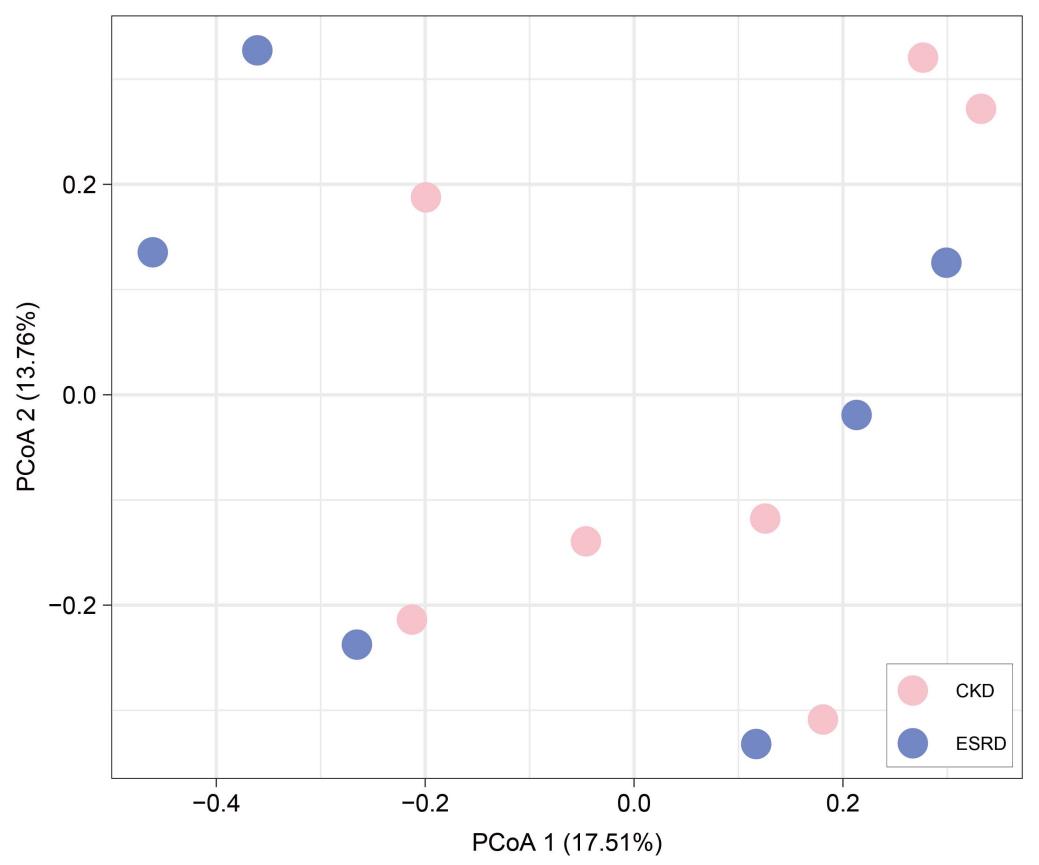


**Supplementary Figure 2**. PCoA presented the differences within group and between CKD and ESRD group.


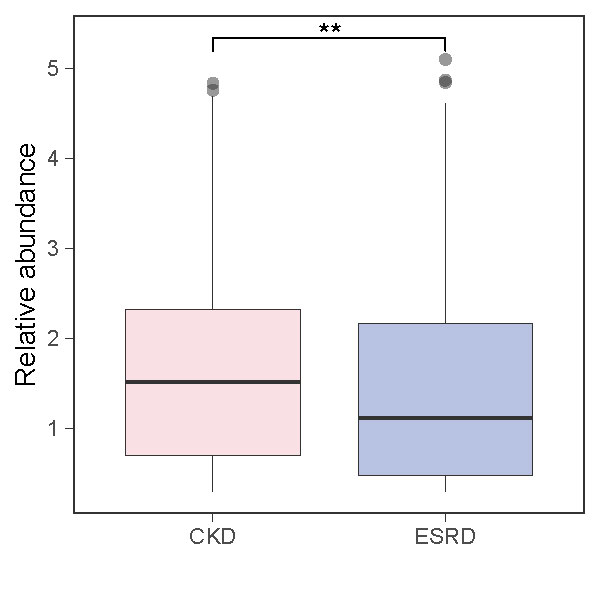


**Supplementary Figure 3.** Boxplot shown the differences in the relative abundance of OTUs from heatmap of Circular maximum likelihood phylogenetic tree between the CKD and ESRD groups. (Wilcox Test, ** *P* < 0.001)


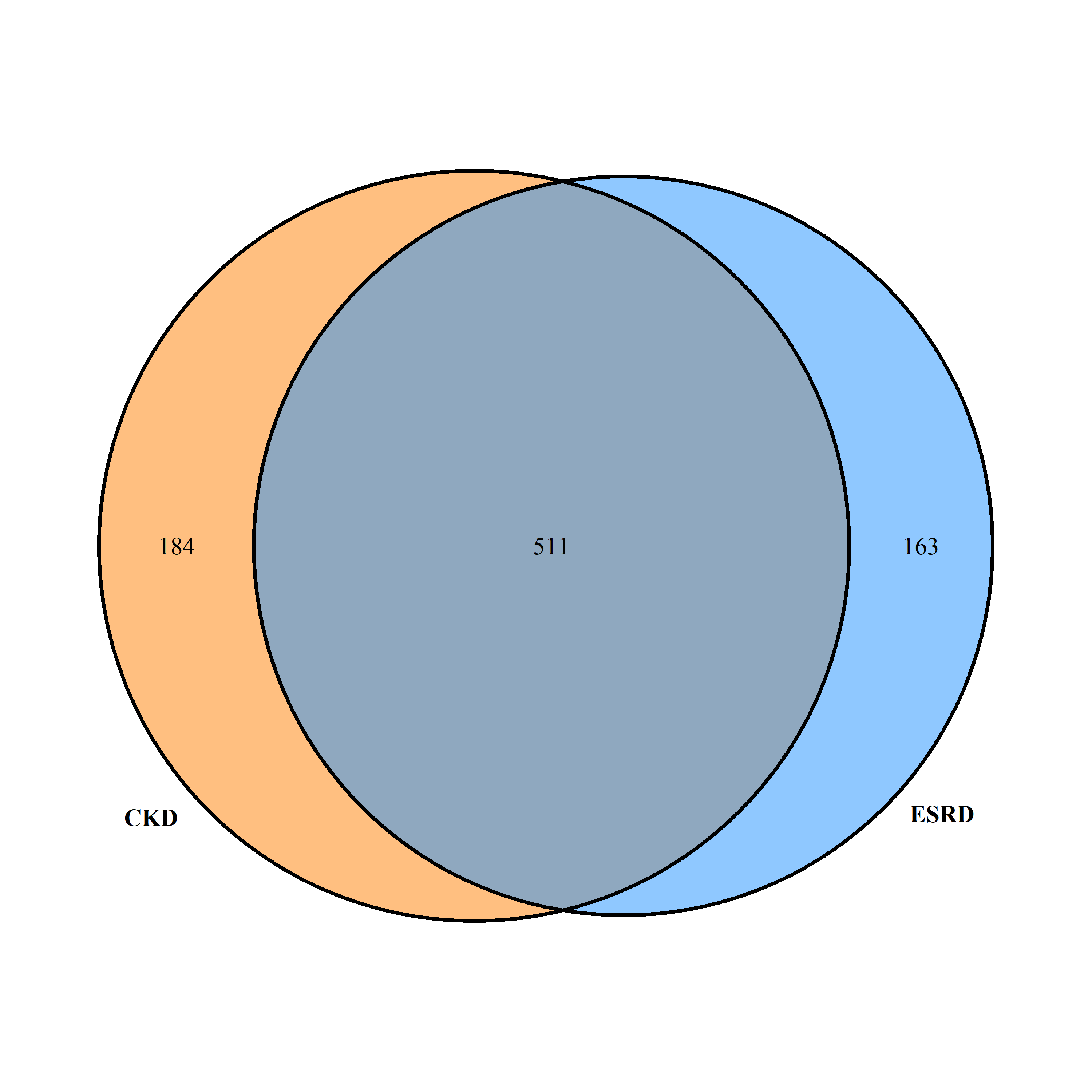


**Supplementary Figure 4.** Venn plot shows the common or specific genera between CKD and ESRD group.


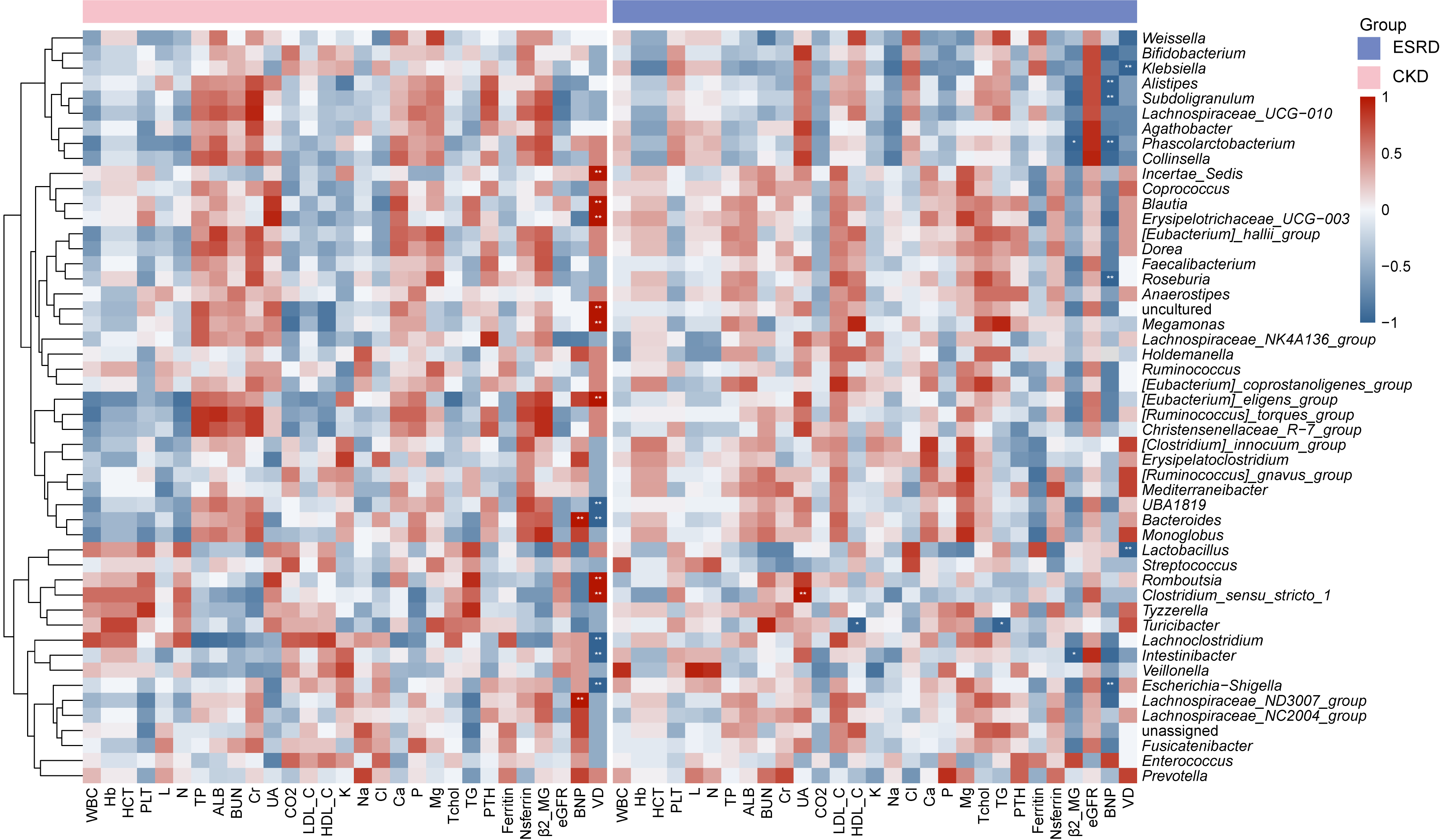


**Supplementary Figure 5**. The correlation between genus relative abundance and environmental factors was calculated by Spearman. For the adjustment for multiple tests, *P*-values are adjusted by the Benjamini and Hochberg correction method. The color of the heat map indicates the correlation coefficient red represents positive correlation and blue represents negative correlation. (* *P*-value <0.05, ** *P*-value < 0.01)


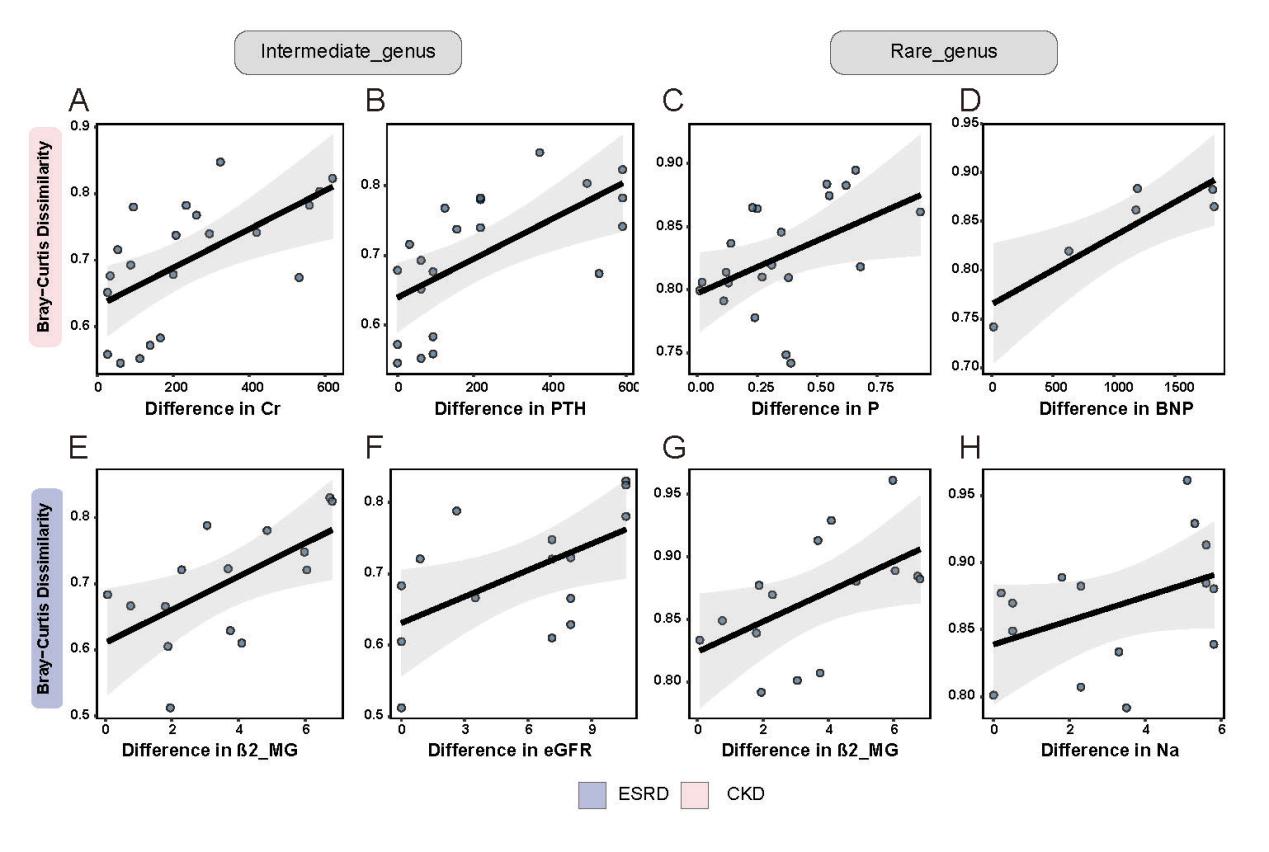


**Supplementary Figure 6.** (A), (B) The scatter plot shown the correlation between the distance of intermediate genera and the distance based on the Cr and PTH. (C), (D) The scatter plot shown the correlation between the distance of rare genera and the distance based on the P and BNP. (E), (F) The scatter plot shown the correlation between the distance of intermediate genera and the distance based on the β2_MG and eGFR. (G), (H) The scatter plot shown the correlation between the distance of rare genera and the distance based on the β2_MG and Na.

**Supplementary Figure 7**.
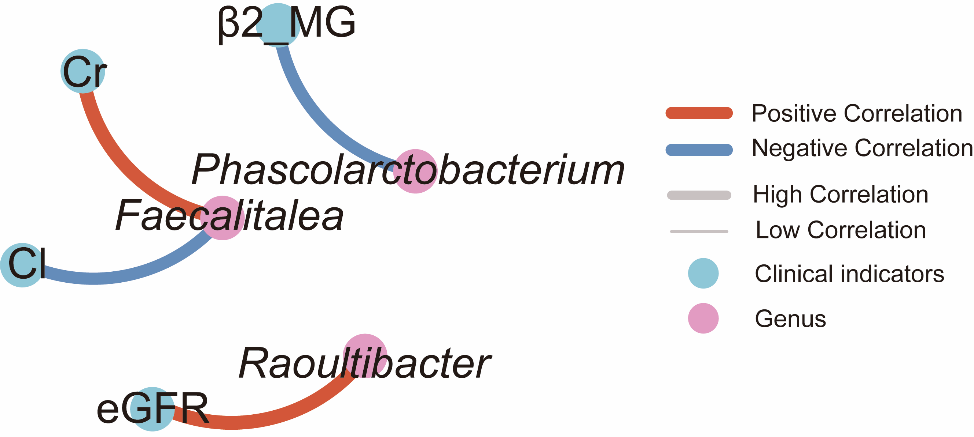
 The correlation analysis of network between clinical indicators and genus with significant difference in relative abundance in the ESRD group. Analysis was performed by Spearman correlation.
